# Oral ezatiostat HCl (Telintra^®^, TLK199) and Idiopathic Chronic Neutropenia (ICN): a case report of complete response of a patient with G-CSF resistant ICN following treatment with ezatiostat, a glutathione S-transferase P1-1 (GSTP1-1) inhibitor

**DOI:** 10.1186/1756-8722-4-43

**Published:** 2011-11-02

**Authors:** Roger M Lyons, Sharon T Wilks, Shelby Young, Gail L Brown

**Affiliations:** 1 Cancer Care Centers of South Texas, US Oncology, San Antonio, TX 78229, USA; 2 Telik Inc., Palo Alto, CA 94304, USA

**Keywords:** idiopathic chronic neutropenia, ezatiostat

## Abstract

Idiopathic chronic neutropenia (ICN) describes a heterogeneous group of hematologic diseases characterized by low circulating neutrophil levels often associated with recurrent fevers, chronic mucosal inflammation, and severe systemic infections. The severity and risk of complications, including serious infections, are inversely proportional to the absolute neutrophil count (ANC), with the greatest problems occurring in patients with an ANC of less than 0.5 × 10^9^/L. This case report describes a 64-year-old female with longstanding rheumatoid arthritis who subsequently developed ICN with frequent episodes of sepsis requiring hospitalization and prolonged courses of antibiotics over a 4-year period. She was treated with granulocyte colony stimulating factors (G-CSF) but had a delayed, highly variable, and volatile response. She was enrolled in a clinical trial evaluating the oral investigational agent ezatiostat. Ezatiostat, a glutathione S-transferase P1-1 inhibitor, activates Jun kinase, promoting the growth and maturation of hematopoietic progenitor stem cells. She responded by the end of the first month of treatment with stabilization of her ANC (despite tapering and then stopping G-CSF), clearing of fever, and healing of areas of infection. This ANC response to ezatiostat treatment has now been sustained for over 8 months and continues. These results suggest potential roles for ezatiostat in the treatment of patients with ICN who are not responsive to G-CSF, as an oral therapy alternative, or as an adjunct to G-CSF, and further studies are warranted.

## Background

Idiopathic chronic neutropenia (ICN) is an uncommon heterogeneous hematologic disorder characterized by persistent severe neutropenia leading to life-threatening infections [1]. Granulocyte colony stimulating factor (G-CSF) has been an effective therapy for increasing blood neutrophil levels in these patients, and the corresponding reduced frequency of fevers, inflammation, and infections has resulted in an improved quality of life. Medical management of neutropenia is mainly symptomatic and consists of antibiotic treatment of febrile patients suspected of having bacterial infections. Other therapies of uncertain efficacy include glucocorticoids, lithium, androgenic steroids, immunoglobulins, and plasmapheresis [2-8].

Although alternative treatment approaches such as administration of granulocyte/macrophage-GCF and corticosteroids have been occasionally reported, G-CSF is the generally accepted treatment for the amelioration of neutropenia in ICN. However, there is no consensus for the dose and duration of G-CSF therapy. This is mainly due to the fact that all data for idiopathic neutropenia arise from heterogeneous patient series comprising cases with diverse underlying pathogenetic mechanisms. The decision for the necessity of G-CSF administration, dose, and short- or long-term duration of treatment is individualized on the basis of infection risk and general clinical judgment rather than the ANC per se. Another important issue is prevention of osteoporosis in ICN patients. It has been shown that treatment with biophosphates significantly improves osteopenia/osteoporosis in these patients. The beneficial effect of the treatment is associated with a reduction in serum levels of IL-1β and TNF-α and, occasionally, with amelioration of neutropenia, substantiating the important role of these inflammatory cytokines in the pathophysiology of ICN [9].

Most patients respond to daily subcutaneous administration of G-CSF; however, a subgroup of patients do not respond. ICN patients undergoing chronic G-CSF therapy often experience bone and muscle pain as well as thrombocytopenia and splenomegaly complicating their therapy.

Ezatiostat is an investigational agent in development for the treatment of a variety of neoplastic and non-neoplastic hematologic disorders, including myelodysplastic syndrome (MDS), and has demonstrated significant improvement in the induction of growth and differentiation of hematologic precursor stem cells as well as an increase in apoptosis of malignant cells. Ezatiostat is an inhibitor of the enzyme glutathione S-transferase P1-1 (GSTP1-1), a negative regulator of Jun kinase (JNK). Treatment of human cells with ezatiostat leads to the activation of JNK, which promotes the growth and differentiation of hematopoietic stem cell precursors. Ezatiostat treatment has shown significant improvement in neutrophil levels in several clinical trials in MDS [10-15]. We report here a patient with longstanding ICN who achieved a complete and sustained hematologic response following treatment with ezatiostat.

## Case presentation

A 64-year-old female with a history of rheumatoid arthritis (RA) since 1985, treated in the past with a variety of agents, including methotrexate, steroids, gold, Imuran, Enbrel, and Humira. The dose and duration of treatments are not available. The patient had borderline leukopenia and neutropenia documented as early as 2001 but developed a more progressive severe neutropenia in 2007. There was no periodicity or cyclical neutropenia. She did not have splenomegaly. Her bone marrow revealed 20-30% cellularity with mild erythroid hyperplasia and mild myeloid and megakaryocyte hypoplasia. There was non-specific lymphocytosis and no dysplasia. The maturation was orderly, with 27% erythroblasts, 1% myeloblasts, and 30% neutrophils and precursors. She experienced numerous hospitalizations for sepsis as a consequence of her neutropenia, with white blood cell counts in the 2000-3000 range and neutrophils less than 5%, hemoglobin of 12.1 gm/dL, and platelet count of 186,000. Rheumatoid factor (RF) was 67 iu, and cyclic citrullinated peptide IgG antibody (CCP-IgG) was > 250 u. Anti-nuclear antibodies varied between negative and 1:160 with a homogeneous pattern. In the 6 months prior to starting G-CSF, her clinical status deteriorated, with multiple admissions to the hospital, fevers as high as 103.8°F, non-healing perineal ulcers, and decubitus ulcers requiring treatment with broad-spectrum intravenous antibiotics. Fevers frequently recurred within 4 days following cessation of parenteral antibiotics, necessitating their resumption. She also suffered from dysphagia and underwent gastrointestinal evaluation with endoscopies, which showed only non-specific gastritis.

She was found to have a small clone of T-gamma lymphocytes (0.8%) in the bone marrow and peripheral blood (confirmed by PCR), but did not respond to steroid pulses or cyclosporine. She had transient moderate renal dysfunction, with a creatinine rising to 3.2 before returning to normal in 4 weeks after cessation of cyclosporine. She was then treated with PEG-filgrastim without response and thereafter with G-CSF at doses as high as 600 mcg/day for 3 months. Following an initial response to G-CSF with some wound healing, her neutropenia became intermittently more severe. Two methylprednisolone dose packs were given during an arthritis flare one month before the study observation period. She was experiencing grade 4 neutropenia and agreed to participate in a phase 2 clinical trial of ezatiostat (Telintra, TLK199). Her median screening absolute neutrophil count (ANC) prior to entering the 2-month observation period was 0.18 × 10^9^/L. The patient received RA treatment while on study, including fish oil, hydroxychloriquine at the start of cycle 7, and acetaminophen or ibuprofen as needed. She received ezatiostat at 2000 mg orally, daily for 21 days of a 28-day cycle, and has continued on ezatiostat therapy. Initially, G-CSF was continued along with the ezatiostat, but tapered and stopped by the end of cycle 2. Her ANC continued to respond after cessation of G-CSF for 8 months and continues.

**Figure 1:**
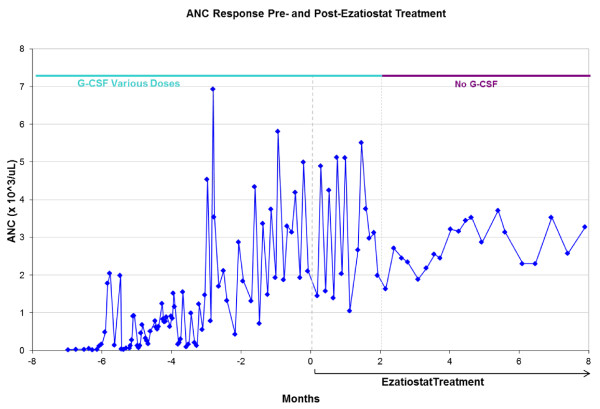
**Absolute neutrophil count showed an inadequate response to G-CSF and ANC response to ezatiostat treatment**.

Her post-ezatiostat treatment median ANC was 3.1 × 10^9^/L. Figure 1 shows the striking improvement in ANC counts beginning with ezatiostat treatment initiation and continuing to respond when G-CSF use was stopped. Figure 1 shows the inadequate ANC response to G-CSF during the baseline run-in period (ANC of .1-.2 × 10^9^/L), as well as post-ezatiostat improvement in ANC counts. Her complete blood counts have remained normal, with an ANC ranging between 1.60-3.75 × 10^9^/L. After 11 months' participation in the study and 7 months of ezatiostat treatment, the T-gamma clone could no longer be identified by flow cytometry but persisted by PCR evaluation. RF had normalized (7 iu) but CCP-IgG remained > 250 u. She has shown remarkable clinical improvement with complete clearing of her arthritic symptoms and with an increase of her Karnofsky performance status from 50% to 90%, and she remains in complete remission from ICN. Accompanying the improvement in her ANC, she experienced a persistent infection-free period and has not required hospitalizations or antibiotic therapy since her ANC improvement. This patient has tolerated ezatiostat well, reporting only grade 1 nausea, abnormal urine and stool odor, and a metallic taste.

## Discussion

Ezatiostat, a glutathione analog, has shown significant effects on growth and differentiation of human bone marrow progenitor stem cells in several preclinical models of myelopoiesis [16,17]. Ezatiostat causes myeloproliferation and differentiation of normal hematopoietic stem cell precursors and apoptosis of malignant cells by disrupting the GSTP1-1/JNK signaling pathway. The mechanism of action of ezatiostat is through the inhibition of GSTP1-1 that results in activation of the JNK pathway. GSTP1-1 has been shown in biochemical assays to be a negative regulator of JNK [18] as well as ERK2. Treatment with ezatiostat in NIH3T3 cells has shown that ezatiostat disrupts the binding of GSTP1-1 to both JNK and ERK2, leading to their activation. Ezatiostat has been evaluated in multiple phase 1 and phase 2 clinical trials in MDS, a syndrome characterized by ineffective hematopoiesis presenting with anemia and, in some cases, neutropenia and thrombocytopenia. Ezatiostat is the first inhibitor of GSTP1-1 that was shown to cause a clinically significant and sustained reduction in red-blood-cell transfusions, transfusion-independence, and multilineage hematologic improvement responses in MDS. Recently, results of a phase 2 multicenter study that evaluated ezatiostat in MDS were reported where ezatiostat was shown to be an active agent with multilineage responses that was well-tolerated in the MDS population [5-7].

We report this unique case of a patient with longstanding ICN who had an inadequate response to G-CSF therapy and required multiple hospitalizations for recurrent infections resulting from her chronic grade 4 neutropenia. This patient received therapy with oral ezatiostat and has shown an impressive hematologic response that is durable and continues. This suggests a potential role for this agent, given its novel mechanism of action, in the treatment of patients with ICN. The oral formulation is potentially of interest for these patients with ICN, since flexibility in dosing and patient convenience in the treatment of a chronic disease such as ICN would be preferable to chronic subcutaneous injections of G-CSF.

## Conclusions

Ezatiostat, a GSTP1-1 inhibitor, activates JNK, promoting the growth and maturation of hematopoietic progenitor stem cells. This case highlights an important observation that ezatiostat produced a striking and sustained hematologic response in ANC in an ICN patient who had an inadequate response to G-CSF. Following 8 cycles of treatment with ezatiostat, she has continued to show remarkable improvement, with an ongoing complete remission from ICN without further G-CSF or need for antibiotics. Her symptomatic improvement has been dramatic. The role of ezatiostat in the suppression of her arthritic symptoms is unclear but may indicate an observation for further evaluation in patients with RA. Additionally, since ezatiostat is an oral agent, it offers ICN patients who require chronic therapy the convenience and flexibility of oral dosing. The results of this case report suggest a potential role for ezatiostat in the treatment of patients with ICN who are not adequately responsive to G-CSF or as an adjunct to G-CSF.

## Consent statement

Written informed consent was obtained from the patient when she was enrolled on this IRB-approved ezatiostat randomized phase 2 ICN trial. A copy of this informed consent, which includes permission for publication, is available to the Editors upon request. Any information that could identify our subject has been withheld.

## Competing interests

Roger Lyons received consulting research support from Amgen, Alexion, Novartis, Celgene, Incyte, and Telik, Inc. Sharon Wilks has no competing interests. Gail Brown and Shelby Young are employed by Telik, Inc.

## Authors' contributions

GB and SY designed the research protocol. RL treated the patient and collected the data. SW referred patient to RL and provided past medical history. GB, RL, and SY wrote the paper. All authors read and approved the final manuscript.

## Statement of Prior Presentation

This case report has not been previously presented.
